# Effects of SAHA and EGCG on Growth Potentiation of Triple-Negative Breast Cancer Cells

**DOI:** 10.3390/cancers11010023

**Published:** 2018-12-27

**Authors:** Kayla A. Lewis, Harrison R. Jordan, Trygve O. Tollefsbol

**Affiliations:** 1 Department of Biology, University of Alabama at Birmingham, 1300 University Blvd, Birmingham, AL 35294, USA; klalewis@uab.edu (K.A.L.); hjordan@uab.edu (H.R.J.); 2 School of Nursing, University of Alabama at Birmingham, 1701 University Blvd, Birmingham, AL 35294, USA; 3 Comprehensive Cancer Center, University of Alabama at Birmingham, 1802 6th Avenue South, Birmingham, AL 35294, USA; 4 Comprehensive Center for Healthy Aging, University of Alabama at Birmingham, 1530 3rd Avenue South, Birmingham, AL 35294, USA; 5 Nutrition Obesity Research Center, University of Alabama at Birmingham, 1675 University Blvd, Birmingham, AL 35294, USA; 6 Comprehensive Diabetes Center, University of Alabama at Birmingham, 1825 University Blvd, Birmingham, AL 35294, USA

**Keywords:** Mesenchymal-to-epithelial transition, breast cancer, DNMT inhibitors, HDAC inhibitors, phytochemicals, microRNA, cancer epigenetics

## Abstract

Triple-negative breast cancer comprises approximately 15–20% of all breast cancers diagnosed and is nearly twice as common in black women than white women in the United States. We evaluated the effects of two epigenetic-modifying compounds on markers of growth potential in several triple-negative breast cancer cell lines. Suberoylanilide hydroxamic acid (SAHA), a histone deacetylase (HDAC) inhibitor currently used in the treatment of cutaneous T cell lymphoma, was administered to triple-negative breast cancer cells alone or in combination with epigallocatechin-3-gallate (EGCG), a DNA methyltransferase (DNMT) inhibitor isolated from green tea. The compounds affected the expression of oncogenic miR-221/222 and tumor suppressors, p27 and PTEN, in addition to estrogen receptor alpha (ERα). E-cadherin expression was increased while N-cadherin was decreased, indicating a more epithelial phenotype. In addition, the activity of DNMTs was diminished with the treatments, and there was a significant enrichment of AcH3 within the promoter of *p27* and *PTEN*, suggesting a role of epigenetic mechanisms for the aforementioned changes. These results translated to reduced migration of the triple-negative breast cancer cells with the treatments. Together, these findings support the role of SAHA and EGCG in limiting growth and proliferation of breast cancer cells.

## 1. Introduction

Triple-negative breast cancer (TNBC) accounts for approximately 15–20% of all diagnosed breast cancers. Most TNBC cases are basal-like, and are associated with a poorer short-term prognosis, women of African descent, and BRCA1 mutations [1]. Basal-like breast tumors are named due to the similarity of their appearance to the outer, or basal, cells surrounding the mammary ducts. These are biologically more aggressive. Studies have shown that TNBC patients have higher rates of local and distant recurrences within 5 years of initial diagnosis [2]. TNBCs are highly heterogeneous due to their classification on three missing growth factor receptors (estrogen receptor (ERα), progesterone receptor (PR), human epidermal growth factor 2 receptor (HER2)); tumor heterogeneity can account for differing responses to the same chemotherapies [3].

Because of the heightened risk of recurrence in TNBC patients, it is imperative to understand the molecular mechanisms behind recurrence [2]. MicroRNAs (miRNAs) are generally 18–24 nucleotides in length, and are capable of modulating a suite of genes, typically by binding the 3′ UTR, preventing translation and tagging the transcript for degradation [4]. miR-221/222 are two miRNAs that are found on the X chromosome as a gene cluster. Because of this, they are typically transcribed together, despite varying expression levels in cells [5]. TNBCs have elevated levels of miR-221/222, and these miRNAs play a role in the epithelial-to-mesenchymal transition (EMT) as well as entry into the S-phase of the cell cycle [6]. Some of their primary targets are *p27*, *ERα*, and *PTEN* mRNA, among others. Previous studies have shown that by inhibiting miR-221/222, ERα expression and tamoxifen sensitivity are restored, there is a decrease in cell growth, and there is an increase in apoptosis [7]. Recent studies have demonstrated that miR-221/222 also promote cancer stem-like cell properties, migration, and invasion, through targeting PTEN, thereby constitutively activating Akt/NF-κB/COX-2 [8].

The field of epigenetics has provided a mechanism for overcoming the variances found in TNBCs. Stirzaker et al. demonstrated in their methylome sequencing of TNBC tumors that there were three distinct clusters associated with hypermethylation based on prognosis [9]. By modifying the TNBC epigenome, it is possible to have a more universal treatment for TNBCs, despite the overall heterogeneity. The use of epigenome-modifying compounds shows promise and potential in the treatment of TNBC in addition to other cancers. Suberoylanilide hydroxamic acid (SAHA) is a histone deacetylase inhibitor that is approved by the FDA to treat cutaneous T cell lymphoma and has shown much promise in treating varying cancers in combination with other chemotherapeutic agents [10,11]. Very little has been studied with respect to metastasis and SAHA, though, in combination with other treatments. Luu et al. demonstrated in their Phase II clinical trial that SAHA had limited potential when administered alone as a metastatic breast cancer chemotherapeutic [12].

Dietary phytochemicals also have the ability to modulate the cancer epigenome [13]. Our research group has performed extensive investigation into the roles of phytochemicals and the cancer epigenome. Epigallocatechin-3-gallate (EGCG) is a green tea polyphenol (GTP) that has already been demonstrated to have anticancer properties through its role as a DNA methyltransferase (DNMT) inhibitor by epigenetically modulating *hTERT* and *ERα* expression [14,15,16]. Despite these results, many of the concentrations used in other studies are not physiologically achievable by diet alone.

Our current findings support the roles SAHA and EGCG have together in reducing the growth potential of TNBC. We have shown that in three TNBC cell lines, there is an overall decrease in TNBC cellular viability with SAHA and EGCG, alone and in combination. Despite this consistent and significant decrease in viability, there are differences among cell types in the molecular mechanisms associated with this increase in cell death, including *miR-221/222* expression and several of their associated downstream targets. These results are also associated with a decrease in cellular migration and the mesenchymal cell marker, *N-cadherin*, with an increase in *E-cadherin*.

## 2. Results

### 2.1. SAHA and EGCG Reduce Cellular Viability and Limit Growth in Four Breast Cancer Cell Lines

**Figure 1:**
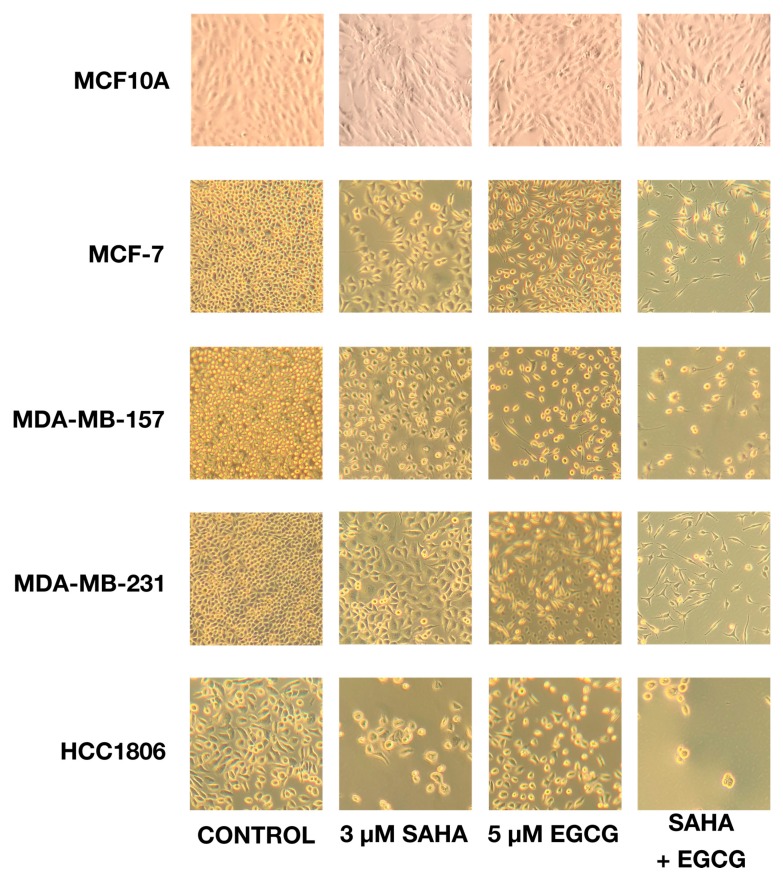
SAHA and EGCG diminish cell density when seeded on a 6-well plate. Cells (2 × 10^5^) were plated on a 6-well plate and treated with SAHA and EGCG alone or in combination (3 μM SAHA + 5 μM EGCG) for 72 h. SAHA and EGCG administered singly were able to reduce the cell density, but not as much as the combination of 3 μM SAHA and 5 μM EGCG. With this combination treatment, not only were there fewer cells present on the plate, but the cells that were visible appeared to be more fibroblastic, suggesting a more highly differentiated state. There was no apparent change in cell density with the treatments in the MCF10A cells. All photos were taken at 100× magnification.

A cell density assay demonstrated the direct effects that SAHA and EGCG had on the growth of cancer cells. With the combination treatment, SAHA and EGCG decreased the density of cells on a 6-well plate (Figure 1), and the remaining cells had a more fibroblastic, or spindle-shaped, appearance, suggesting a higher level of differentiation with the treatment of SAHA and EGCG. Single doses of SAHA and EGCG were also capable of decreasing cellular density, but to a lesser degree than the optimal combination of 3 μM SAHA and 5 μM EGCG. There did not appear to be any negative effects on the MCF10A cells, and we proceeded to focus on the MCF-7, MDA-MB-157, MDA-MB-231, and the HCC1806 cell lines for the remainder of our studies. The HCC1806 cell line appeared to be most susceptible to this treatment, which could be due to the elevated time for population doubling in comparison to the MDA-MB-231 cell line. Determination of optimal concentrations of SAHA and EGCG alone or in combination are shown in Figure S1.

### 2.2. SAHA and EGCG Decrease the Activity of DNMTs

**Figure 2:**
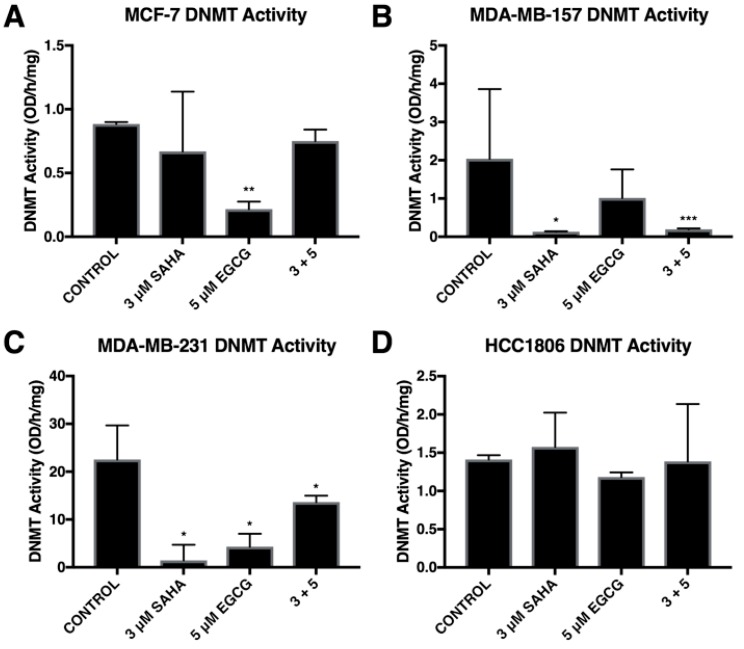
Overall DNMT activity was modulated by SAHA and EGCG. (**A**) MCF-7 cells were treated for 72 h with the indicated compounds. DNMT activity was measured with an ELISA-based assay. (**B**–**D**) The same was done in the MDA-MB-157, MDA-MB-231, and HCC1806 cell lines. All experiments were repeated three times. Error bars represent standard error of the mean (SEM); * *p* < 0.05, ** *p* < 0.01, *** *p* < 0.001.

Combinatorial SAHA and EGCG treatments at optimal concentrations decreased the activity of DNMTs in two triple-negative breast cancer cell lines, MDA-MB-157 and MDA-MB-231, and the ERα-positive MCF-7 cell line (Figure 2A–C). In addition, we found that SAHA was capable of significantly decreasing DNMT activity in the MDA-MB-157 cell line (Figure 2B), as previously reported [17]. We also found this to occur in the MDA-MB-231 cells although it did not significantly decrease DNMT activity in MCF-7 and HCC1806 breast cancer cells (Figure 2A,D).

### 2.3. miR-221/222 Expression is Reduced with the Combination Treatment of SAHA and EGCG

**Figure 3:**
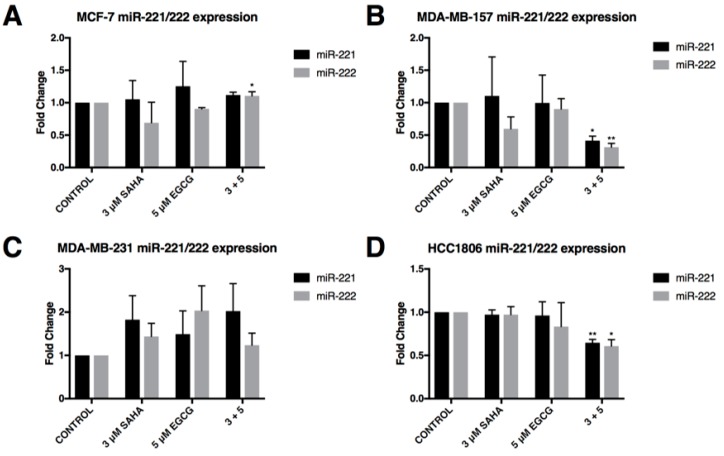
In combination, SAHA and EGCG significantly reduce the expression of miR-221/222 in two triple-negative breast cancer cell lines. (**A**) qRT-PCR was completed using MCF-7 cells after 3-day treatments with the indicated compounds using *miR-221* and *miR-222* primers (*n* = 3). *RNU6B* was used for comparison; (**B**–**D**) the same was done in MDA-MB-157, MDA-MB-231, and HCC1806 cells (*n* = 3). Error bars represent standard error of the mean (SEM); * *p* < 0.05, ** *p* < 0.01.

MCF-7 breast cancer cells have low endogenous expression of *miR-221/222* in comparison to the triple-negative breast cancer cell lines in which estrogen receptor, α, is repressed in part by miR-221/222 [18]. We found limited changes in the expression of *miR-221/222* in the MCF-7 cell line (Figure 3A). By contrast, the MDA-MB-157 and HCC1806 cell lines experienced a significant decrease in *miR-221/222* expression with the combination of SAHA and EGCG (Figure 3B,D). The MDA-MB-231 cells appeared to have an upward trend in the expression of *miR-221/222* with the treatment of SAHA and EGCG, though this was not significant (Figure 3C).

**Figure 4:**
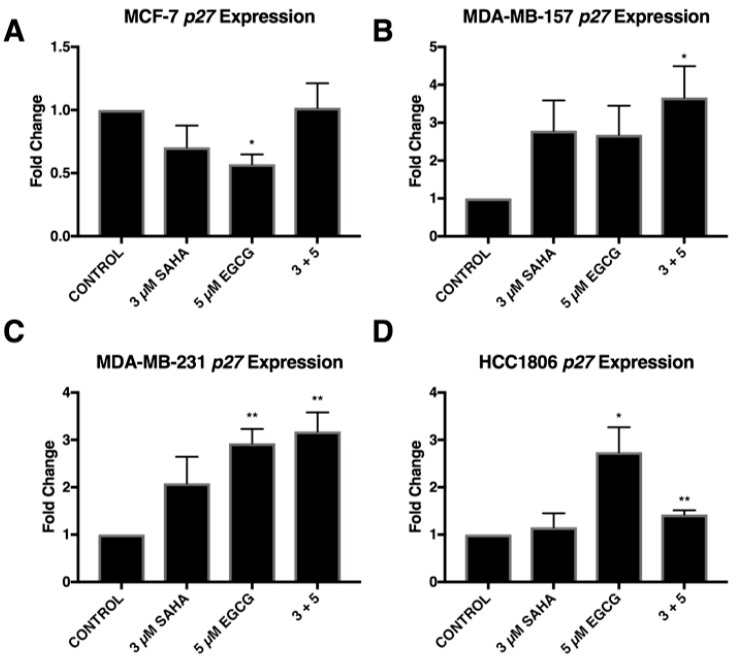
SAHA and EGCG in combination are capable of restoring *p27* expression in three triple-negative breast cancer cell lines. (**A**) qRT-PCR using *p27* primers was performed for MCF-7 cells after 3-day treatments with the indicated compounds (*n* = 3). *GAPDH* was used for comparison; (**B**–**D**) the same was done in the MDA-MB-157, MDA-MB-231, and HCC1806 cells (*n* = 3). Error bars represent the standard error of the mean (SEM); * *p* < 0.05, ** *p* < 0.01.

**Figure 5:**
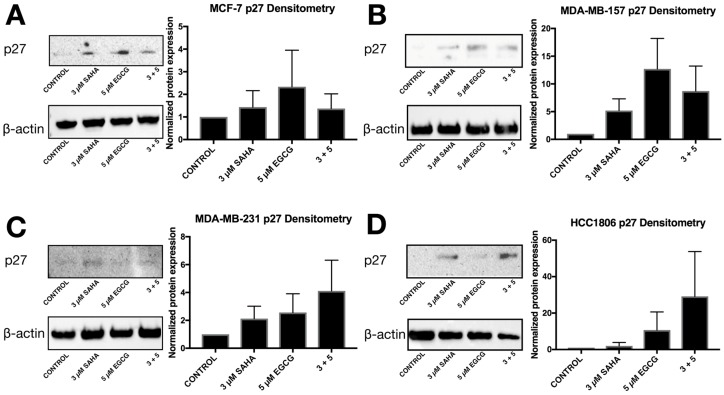
The overall protein level of p27 is restored with the combination of SAHA and EGCG. (**A**) Western blot protein analysis using p27 antibodies was performed using MCF-7 cells after 3-day treatments with the indicated compounds (*n* = 3) (Cell Signaling Technologies, Danvers, MA, USA). β-actin was used for comparison. Densitometry analysis was completed using ImageJ software (*n* = 3). (**B**–**D**) The same was done in MDA-MB-157, MDA-MB-231, and HCC1806 triple-negative cell lines. Pictures are representative of the replicates; densitometry is an average of all three replicates. The same blots were probed multiple times, explaining the duplication of β-actin between figures.

**Figure 6:**
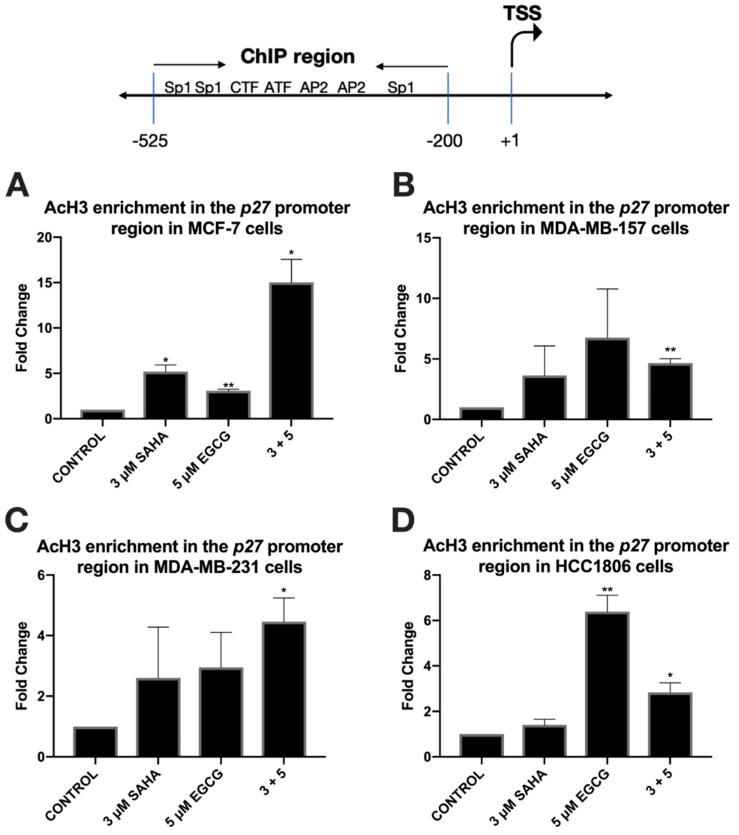
Acetylated histone H3 is enriched in the promoter region of *p27* with the combination treatment of SAHA and EGCG. (**A**) ChIP qRT-PCR analysis using AcH3 antibodies (Millipore Sigma, Burlington, MA, USA) and p27 promoter primers (Integrated DNA Technologies, Coralville, IA, USA) was performed with a ChIP kit (Abcam, Cambridge, MA, USA) in MCF-7 cells after 3-day treatments with the indicated compounds (*n* = 3). (**B**–**D**) The same was done in the MDA-MB-157, MDA-MB-231, and HCC1806 triple-negative cell lines. Histone H3 antibody (Abcam) was used as a positive control for comparison.

To further explore the effects of reduced miR-221/222 expression, qRT-PCR and Western blot analysis were performed on three direct targets of miR-221/222: p27, PTEN, and estrogen receptor α (ERα). The expression of *p27* was significantly increased in all three triple-negative cell lines with the combination of SAHA and EGCG (Figure 4). EGCG alone appeared to increase the expression of *p27* to a greater degree than the combination in the HCC1806 cell line, but the change was not found to be significant (Figure 4D). The combination treatment did not affect the expression of *p27* in the MCF-7 cell line, but the treatment with EGCG alone significantly reduced the expression of *p27* (Figure 4A). P27 protein was increased with the combination in all four cell lines (Figure 5). To determine if these effects were due to direct epigenetic alteration, we performed chromatin immunoprecipitation (ChIP) analyses on a key segment of the promoter region of *p27* based on the associated transcription factor binding sites (Figure 6). We analyzed acetylated histone H3 (AcH3), which is associated with active gene transcription, in a region of the *p27* promoter comprising several transcriptional activators and noticed a significant enrichment of AcH3 in all four cell lines with our combination of SAHA and EGCG. We also performed cell cycle analysis after determining a significant restoration of p27 (Supplementary Figure S2). There was an increase in the G1/S phase, with a reduction in the G2 phase in the MCF-7, MDA-MB-157, and MDA-MB-231 cell lines (Figure S2A–C). Interestingly, there was an increase in the G2 phase in the HCC1806 cells with the treatment of SAHA alone and in conjunction with EGCG, suggesting another potential mechanism associated with the decrease in cellular viability (Figure S2D).

**Figure 7:**
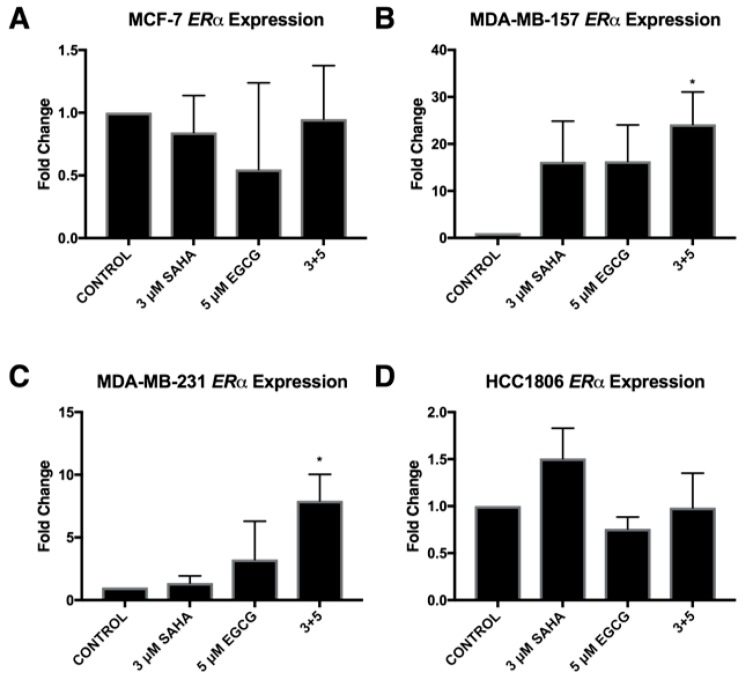
Estrogen receptor alpha (*ERα*) is restored in three TNBC cell lines with the combination of SAHA and EGCG. (**A**) MCF-7 cells were treated for 72 h with the indicated compounds. qRT-PCR was completed using *ERα* primers. (**B**–**D**) The same was completed in MDA-MB-157, MDA-MB-231, and HCC1806 cell lines (*n* = 3). Error bars represent standard error of the mean (SEM); * *p* < 0.05.

**Figure 8:**
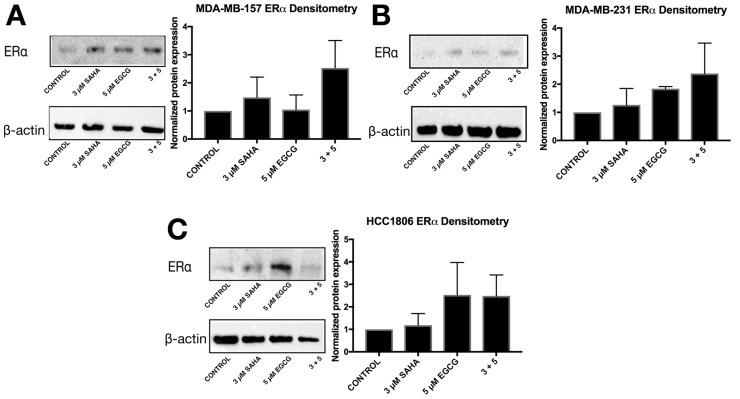
SAHA + EGCG upregulated estrogen receptor alpha (ERα) in all three TNBC cell lines. (**A**) After 72 h of treatment with SAHA and/or EGCG, protein was extracted from MDA-MB-157 cells. Cells were probed with ERα primary antibodies (Santa Cruz, Dallas, TX, USA) before imaging. β-actin was used as the control. (**B**,**C**) The same was done in MDA-MB-231 and HCC1806 cell lines. Pictures are representative while densitometry analysis is an average of all replicates (*n* = 3). The same blots were probed multiple times, explaining the duplication of β-actin between figures.

**Figure 9:**
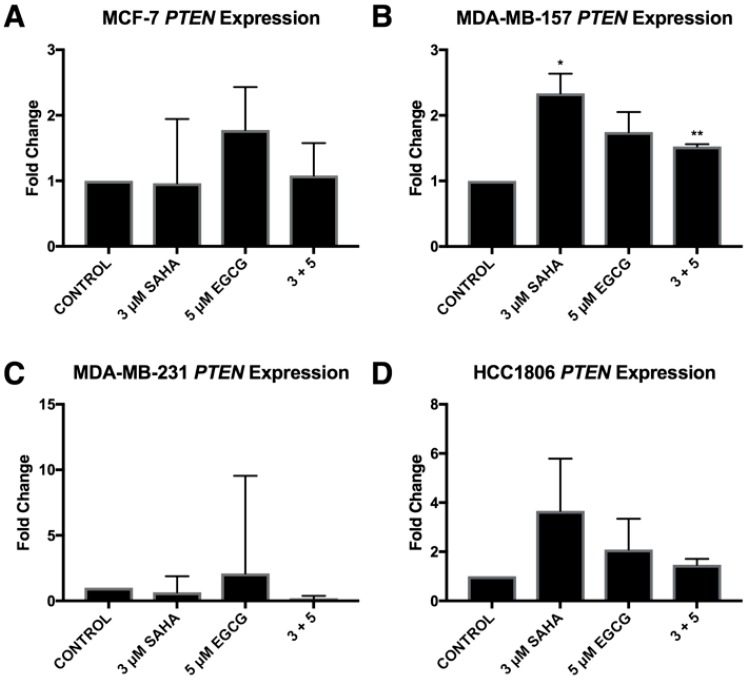
*PTEN* gene expression was only altered by SAHA and EGCG in the MDA-MB-157 cell line. (**A**) MCF-7 cells were treated with the indicated compounds for 72 h. qRT-PCR was completed using *PTEN* primers. (**B**–**D**) The same was done in the MDA-MB-157, MDA-MB-231, and the HCC1806 cell lines (*n* = 3). Error bars represent standard error of the mean (SEM); * *p* < 0.05; ** *p* < 0.01.

**Figure 10:**
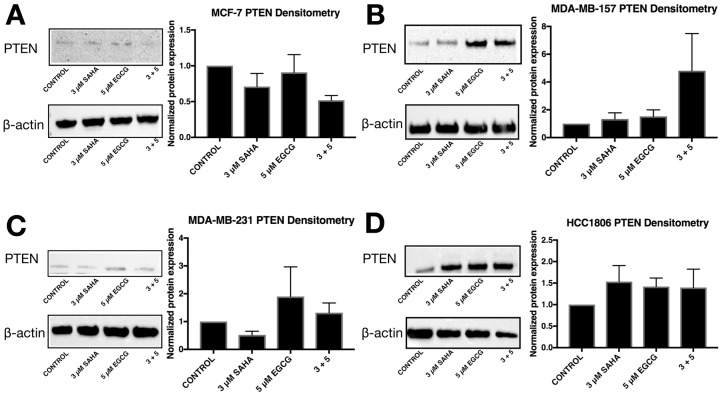
SAHA and EGCG induce changes in PTEN protein levels in three TNBC cell lines. (**A**) PTEN protein levels are reduced in the ERα-positive MCF-7 cells. The image is representative while quantification is indicative of the averages of three different blots. (**B**–**D**) The amount of PTEN protein was increased in the MDA-MB-157, MDA-MB-231, and the HCC1806 TNBC cell lines. The images are representative while quantification is indicative of the averages of three different blots. The same blots were probed multiple times, explaining the duplication of β-actin between figures.

**Figure 11:**
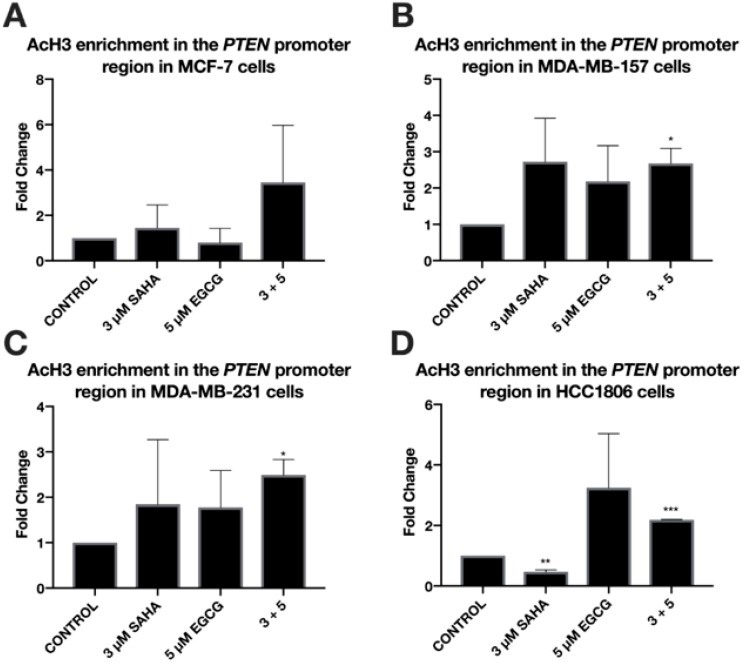
Enrichment of acetylated histone H3 within the *PTEN* promoter is associated with the treatment of SAHA and EGCG in three TNBC cell lines. (**A**) ChIP qRT-PCR was performed in MCF-7 breast cancer cells utilizing an AcH3 antibody (Millipore Sigma) and an H3 antibody (Abcam) for a positive control. Primers for a region of the PTEN promoter were used (Integrated DNA Technologies) in conjunction with the ChIP kit (Abcam). Cells were treated for 3 days with the indicated compounds prior to performing ChIP (*n* = 3). (**B**–**D**) The same was done in MDA-MB-157, MDA-MB-231, and HCC1806 TNBC cell lines (*n* = 3). * *p* < 0.05; ** *p* < 0.01; *** *p* < 0.001

ERα mRNA levels were significantly increased in the MDA-MB-157 and MDA-MB-231 cell lines with the combinatorial SAHA and EGCG treatments at optimal concentrations (Figure 7B,C). There was no significant change in the MCF-7 or HCC1806 cell lines (Figure 7A,D). Interestingly, ERα protein was increased in all three TNBC cell lines (Figure 8). PTEN mRNA was only upregulated in the MDA-MB-157 cell line (Figure 9), while PTEN protein was restored in all three TNBC cell lines (Figure 10). To determine if there were any epigenetic alterations to the *PTEN* promoter region, we performed chromatin immunoprecipitation (ChIP) analysis on a region of the *PTEN* promoter (Figure 11). We noted a significant increase in acetylated histone H3 with the combination treatment in all three triple-negative cell lines, with no significant changes in the MCF-7 cells. Primers were chosen from the previous work of Shen et al. [19].

### 2.4. Migratory Capabilities are Reduced with the Combination of SAHA and EGCG

**Figure 12:**
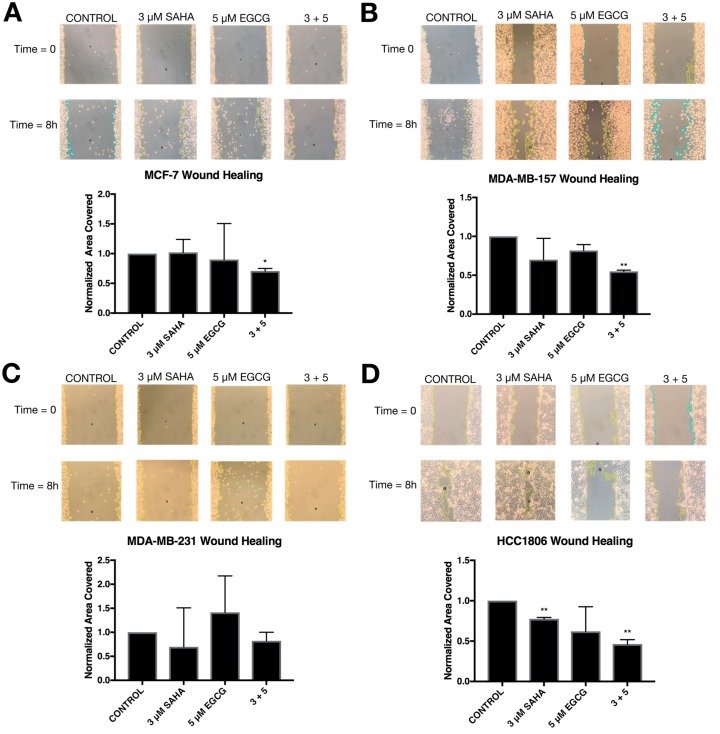
SAHA and EGCG are capable of inhibiting migration in MCF-7, MDA-MB-231, and HCC1806 breast cancer cells. (**A**) MCF-7 cells were treated with the indicated compounds for 72 h and then scratched using a 1000 μL pipette tip. The culture media was replaced, and the cells were given 8 h to migrate. (**B**–**D**) The same procedure was completed with the MDA-MB-157, MDA-MB-231, and HCC1806 cells (*n* = 3). Error bars represent standard error of the mean (SEM); * *p* < 0.05, ** *p* < 0.01. All photos were taken at 100× magnification.

**Figure 13:**
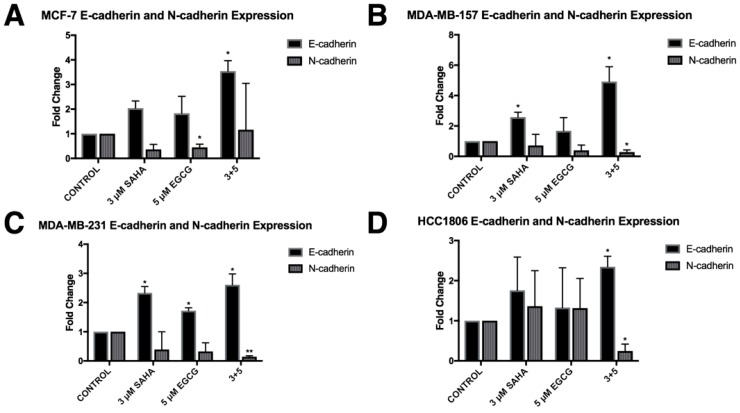
The expression of E-cadherin was induced with SAHA and EGCG while the expression of N-cadherin was reduced. (**A**) MCF-7 cells were treated for 72 h with the indicated compounds. qRT-PCR was completed using *E-cadherin* or *N-cadherin* primers. *N-cadherin* was significantly reduced by EGCG alone. (**B**–**D**) The same was completed in MDA-MB-157, MDA-MB-231, and HCC1806 cell lines (*n* = 3). Error bars represent standard error of the mean (SEM); * *p* < 0.05, ** *p* < 0.01.

**Figure 14:**
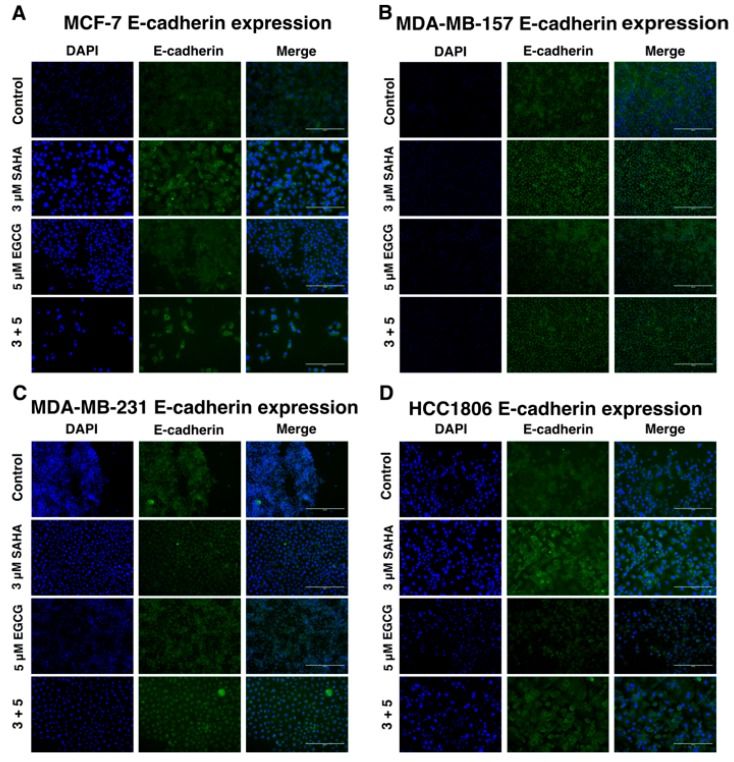
E-cadherin expression is increased on the cell surface with the combination treatment of SAHA and EGCG. (**A**) MCF-7 cells were treated for 72 h with the indicated compounds then fixed with 4% formaldehyde and blocked with 10% fetal calf serum. Anti-E-cadherin antibody (Cell Signaling Technology, Danvers, MA, USA) was applied at a 1:100 dilution overnight then washed with PBST. Fluorescently tagged secondary antibody (Abcam) was applied at a 1:1000 dilution for 30 min before adding mounting media (Thermo Fisher, Waltham, MA, USA) and letting it dry overnight. (**B**–**D**) The same was done in MDA-MB-157, MDA-MB-231, and HCC1806 TNBC cells. Cells were imaged on an EVOS microscope at 20× (*n* = 3).

In order to determine the effects of SAHA and EGCG on cellular migration, a wound healing assay was performed on all four cell lines (Figure 12). There was a significant decrease in migration with the combination of SAHA and EGCG in MCF-7, MDA-MB-157, and HCC1806 breast cancer cell lines. There appeared to be a decrease in migration in the MDA-MB-231 cell line compared to the control, but this was not significant (Figure 10C). The area of the scratch was measured using the MRI Wound Healing Tool macro designed for ImageJ, as cited in recent papers [20,21]. Because of the general decrease in cellular migration, *E-cadherin* and *N-cadherin* levels were quantified (Figure 11). *E-cadherin* was significantly increased in all four cell lines, while *N-cadherin* was significantly decreased in all three TNBC cell lines (Figure 13). EGCG alone was able to decrease *N-cadherin* in the MCF-7 cell line (Figure 13A). We completed immunofluorescence with an anti-E-cadherin antibody and noted an increase in fluorescence in all four cell lines with the combination treatment of SAHA and EGCG (Figure 14).

## 3. Discussion

While dietary phytochemicals have generated much interest in cancer treatment and prevention, many studies utilize concentrations that are not physiologically achievable by diet alone. EGCG has been demonstrated to act as a competitive inhibitor of DNMTs, which can be easily counteracted if there is considerably more substrate available [22]. Because of this, at physiologically relevant concentrations of EGCG, EGCG is a noticeably weaker DNMT inhibitor in comparison to other phytochemicals that have been identified as DNMT inhibitors [23]. Previous studies from our lab have demonstrated that EGCG can induce re-expression of endogenous estrogen receptor α (ERα) in ERα-negative MDA-MB-231 breast cancer cells, but mainly through histone modifications [14]. We demonstrated that this reactivation was primarily through chromatin remodeling and histone acetyltransferase (HAT) activation and was synergistically enhanced when paired with a histone deacetylase inhibitor (TSA). Additional studies have indicated that EGCG can inhibit telomerase activity through epigenetic regulation of the *hTERT* (human telomerase reverse transcriptase) gene [24]. This could be another potential mechanism behind the increase in cell death observed in this study.

**Figure 15:**
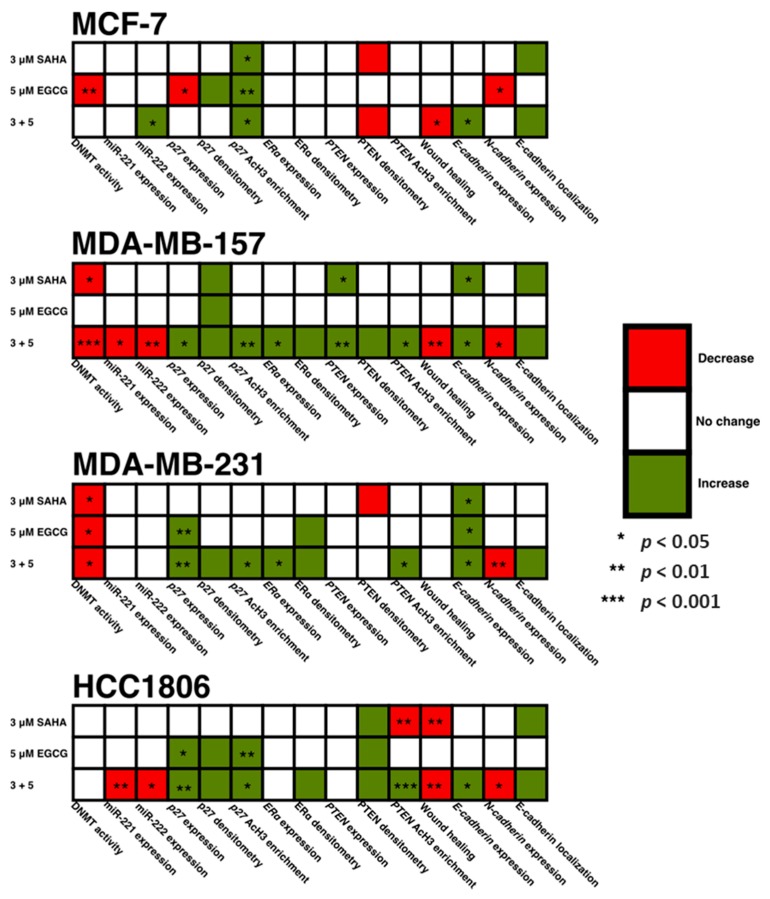
A summative figure visualizing the findings from the present study. Despite all four cell lines demonstrating a decrease in cell density and growth in Figure 1, there is variance in the mechanisms behind the decrease. The heterogeneity of TNBC is easily visualized in this figure, which can represent the importance of epigenetic-based treatment of TNBC.

In the present study, we combined EGCG with the FDA-approved HDAC inhibitor, SAHA, to study growth potentiation in three TNBC cell lines, with an ERα-positive cell line used for comparison. Figure 15 is a summative figure addressing our findings from the present study. With the combination, *miR-221/222* was reduced in the MDA-MB-157 and HCC1806 cell lines. Interestingly we observed an increase in *mir-221/222* expression in the MCF-7 cell line with the combinatorial treatment of SAHA and EGCG. Endogenous expression of *miR-221/222* was low in the MCF-7 cell line, so this increase does not necessarily imply levels equal to that of the TNBC cell lines [18].

Because *miR-221/222* have many targets that result in tumorigenesis, we aimed to investigate the expression of these downstream targets. P27 is a tumor suppressor protein involved in cell cycle progression through the G1/S phase [25]. SAHA and EGCG were capable, in combination, of not only upregulating the expression of *p27* in all three TNBC cell lines, but also the protein levels of p27 in the MCF-7, MDA-MB-157, MDA-MB-231, and HCC1806 cell lines. Loss of p27 in cancer is rarely due to methylation, mainly because of the numerous posttranscriptional modifications that occur [26]. We did note an increase in AcH3 within a region of the *p27* promoter region that is associated with active gene transcription factors. This provides an epigenetic mechanism behind the restoration of p27 expression. Because of these results, we conducted cell cycle analysis on all four cell lines after SAHA and/or EGCG treatment and found a decrease in the G2 phase in the MCF-7, MDA-MB-157, and the MDA-MB-231 cell lines (SF2). Interestingly, though, we found that the HCC1806 cell line incurred an increase in the G2 phase with the incorporation of SAHA. HCC1806 cells are metaplastic and highly refractory to treatment. It has been demonstrated that metaplastic cell lines overexpress the TRIM24 histone acetylation reader. TRIM24 can act as either a tumor suppressor or oncogene depending on the type of cancer and recognizes acetylated H3 lysine 23 (H3K23ac). Previous studies determined that elevated H3K23ac and TRIM24 predict shorter overall survival of breast cancer patients [27,28,29]. Because SAHA acts as an HDAC inhibitor, it is possible that SAHA promoted the presence of H3K23ac in the HCC1806 cell line, allowing TRIM24 to promote the transcription of genes associated with the G2/M phase [30,31].

We provided evidence in this study that a lower concentration of EGCG in combination with SAHA was capable of inducing ERα expression in two out of three TNBC cell lines, with MDA-MB-231 cells exhibiting similar results as our previous studies with EGCG [14]. Additional studies from our laboratory have shown that EGCG in combination with sulforaphane, which is an isothiocyanate found in cruciferous vegetables, directly influence the expression of DNMT1 and HDAC1 and restore the expression of ERα. These results were similar to the effects of DNMT1 and HDAC1 knockdown [16]. ERα protein levels were upregulated in all three TNBC cell lines, but to a lesser degree in the MDA-MB-231 cells. This result corresponds with the overall aggressive nature of the MDA-MB-231 cell line. Although we did not investigate the sensitivity to tamoxifen in our study, the results suggest that SAHA and EGCG, in combination, could restore tamoxifen sensitivity in hormone-resistant and triple-negative breast cancers [16].

The expression of *PTEN* was only significantly upregulated in the MDA-MB-157 cell line. Because *miR-221/222* was not significantly down-regulated in the MDA-MB-231 cell line (Figure 3C), it was not surprising that *PTEN* was not increased with the treatment of SAHA and EGCG [32]. *PTEN* is not suppressed in MCF-7 cells, but it was interesting to note that PTEN expression was not upregulated with the combination treatment (Figure 5A) [33]. Previous studies have demonstrated that the loss of *PTEN* expression is associated with an increase in promoter hypermethylation, with a positive correlation of PTEN and ER or PR, and high PTEN expression was significantly associated with disease-free survival [34,35]. Despite *miR-221/222* suppression in the HCC1806 cell line, *PTEN* was not significantly modulated. The HCC1806 cell line is described as an acantholytic squamous cancer and metaplastic, which is highly refractory to treatment [36]. In all of the described experiments, HCC1806 cells exhibited unique results in comparison to the MDA-MB-157 and MDA-MB-231 cell lines. Our Western blot analyses demonstrated an increase in PTEN protein levels in all three TNBC cell lines (Figure 4). We conducted ChIP analysis of the *PTEN* promoter region encompassing an ATF binding site, which is a transcriptional activator [19]. We analyzed AcH3 and noted its significant enrichment in all three TNBC cell lines, but not in the MCF-7 cell line.

Because ERα is an epithelial cell marker, we were interested in determining the effects of SAHA and EGCG on the epithelial-to-mesenchymal transition (EMT). Bouris et al. previously demonstrated that knocking down ERα in MCF-7 cells resulted in phenotypic changes and changes in gene and protein expression of markers of EMT [37]. The wound healing assay provided phenotypic evidence that SAHA and EGCG limited the ability of the cell lines to migrate (Figure 10). Although there was not a significant decrease in migration in the MDA-MB-231 cell line, there were clearly more cells in the scratched region that were not detected by the ImageJ software in the single treatments versus the combination treatment (Figure 10C). We believe that these results still support our hypothesis. We then decided to investigate the gene expression of *E-cadherin* and *N-cadherin* to determine if there was an increase in epithelial markers and a decrease in mesenchymal markers with SAHA and EGCG treatments. Our findings indicated that in all four cell lines, there was an increase in *E-cadherin* with the combination, and there was a decrease in *N-cadherin* with the combination treatment in the TNBC cell lines. EGCG alone reduced the expression of *N-cadherin* in the MCF-7 cell line, but not the combination. It has previously been demonstrated that *miR-221/222* down-regulate E-cadherin through targeting of the 3′UTR of TRPS1 (trichorhinophalangeal syndrome type 1), a transcriptional repressor that inhibits EMT through repressing *ZEB2* [38]. E-cadherin has the ability to upregulate p27, so the increase in *E-cadherin* expression with the combination could play a role in the increase in *p27* expression in this paper [26]. We performed immunofluorescence with an anti-E-cadherin antibody on all four cell lines and determined there was a notable increase in cell membrane localization of E-cadherin with the combination treatment.

The current study has provided a basis of support behind the rationale to study combinatorial SAHA and EGCG in more depth with regard to epigenetic mechanisms responsible for the epithelial phenotype discovered in this study. Our results support the roles of SAHA and EGCG in the regulation of *miR-221/222* in addition to DNMT activity. It has previously been established that SAHA and EGCG act as HDAC inhibitors and DNMT inhibitors, respectively [39,40]. Future studies will focus on the epigenetic mechanisms behind the decrease in not only cellular viability, but also migration. We intend to investigate epigenetic modifications in the promoter region of *p27* with the combination of SAHA and EGCG in addition to more experiments associated with metastatic potential, including fibronectin (FN) migration and mammosphere generation. Because of the decrease in cellular viability and migration, we also plan to investigate apoptosis with SAHA and EGCG, including *IAP2*, which is known to promote EMT in TNBC cells [41].

## 4. Methods and Materials

### 4.1. Cell Lines

ERα (+) MCF-7 and ERα (-) MDA-MB-157, MDA-MB-231, and HCC1806 breast cancer cells were selected for this study. MCF10A human mammary epithelial cells were used as a non-cancerous control (ATCC, Manassas, VA, USA).

### 4.2. Chemicals

EGCG (≥ 97% pure, HPLC) and SAHA (≥ 98% pure, HPLC) were purchased from Sigma-Aldrich (St. Louis, MO, USA). The compounds were prepared in dimethyl sulfoxide (DMSO), which was obtained from Sigma-Aldrich and stored at a stock concentration of 100 mM and 25 mM, respectively, at −20 °C.

### 4.3. Cell Culture and Treatment

All cells were cultured as described in the ATCC protocol. MDA-MB-157, MDA-MB-231, and MCF-7 cells were grown in DMEM media (GIBCO). HCC1806 cells were grown in RPMI-1640 medium. MCF10A cells were cultured using DMEM F12 media in addition to 5% donor horse serum, 100 μL of 20 ng/mL EGF, 50 μL of 100 ng/mL cholera endotoxin, 100 μL of 0.05 μg/mL hydrocortisone, 0.292 g of 2 mmol/L L-glutamine, and 5 mL of 100 units/mL penicillin streptomycin. The cells were subcultured at 80%–90% confluence, and maintained in an incubator at 5% CO_2_ with a controlled temperature of 37 °C. After seeding, cells were allowed 24 h to adhere to plates after which they were treated over a one or three-day period with SAHA, EGCG, or both at the indicated concentrations. Treatments were administered every 24 h with fresh media. DMSO was used as a vehicle control.

### 4.4. MTT Analysis

The number of viable cells in each well was estimated by the uptake of the tetrazolium salt, 3-(4,5-dimethylthiazol-2-yl)-diphenyltetrazolium bromide (MTT). MTT was converted to a purple insoluble formazan by a mitochondrial enzyme, which was further dissolved using DMSO. Readings were acquired at 595 nm using a microplate reader (Epoch model, Biotek, Winooski, VT, USA). Cells (2 × 10^3^) were plated and allowed to attach to a 96-well plate, and then treated with the indicated compounds for the indicated time period.

### 4.5. Cell Density Analysis

Cells (2 × 10^5^) were plated and allowed to attach to a 6-well plate, and then treated with the indicated compounds for 72 h. At 72 h, the density of the cells was analyzed using an inverted microscope using a 10 × objective lens and 10 × ocular magnification (Zeiss, Jena, Germany).

### 4.6. Nuclear Extraction

Nuclear extracts were prepared by resuspending cell pellets in 1× NE1 solution mixed with DTT solution and PIC as provided by the EpiQuik™ Nuclear Extraction Kit (Epigentek, Farmingdale, NY, USA). Cells were incubated on ice for 10 min and centrifuged for 1 min at 12,000 rpm. The supernatant was carefully removed, and the pellet was resuspended in NE2 containing DTT and PIC. The extract was incubated on ice for 15 min, sonicated, and centrifuged at 14,000 rpm for 10 min at 4 °C. The supernatant was transferred to a new vial, and the protein concentration of the nuclear extract was quantified with a Bradford protein assay using the Bio-Rad Protein Assay (Bio-Rad; Hercules, CA, USA).

### 4.7. DNMT Activity

DNMT activity was quantified according to the ELISA-based protocol supplied by the EpiQuik™ DNA Methyltransferase Activity/Inhibition Assay Ultra Kit (Epigentek), where the DNMT substrate is coated onto microplate wells and purified nuclear proteins transfer methyl groups from Adomet to methylate the DNA substrate. An anti-5-methylcytosine antibody recognizes the newly methylated DNA, which can be measured by reading the absorbance with a spectrophotometer at 450 nm.

### 4.8. RNA Extraction

Total RNA was extracted by first lysing and homogenizing cells. Ethanol was then added and then loaded into an RNeasy silica membrane spin column from the RNeasy Kit (Qiagen, Valencia, CA, USA). RNA bound the column and was then purified with subsequent washes.

### 4.9. Protein Extraction

Protein extractions were prepared according to the protocol used during the telomerase PCR ELISA (TRAP) kit (Roche applied science, Indianapolis, IN, USA). Cells were centrifuged at 3000× *g* for 10 min after washing with PBS, resuspended in 200 μL Lysis reagent, and incubated on ice for 30 min. Cells were centrifuged at 16,000× *g* for 20 min at 4 °C, and 175 μL of the supernatant were transferred to a new tube and stored at −80 °C until further use.

### 4.10. Reverse Transcription-Polymerase Chain Reaction (RT-PCR)

RT-PCR was used to examine the expression of genes of interest. RNA (10 μL) was reverse-transcribed to cDNA with the iScript™ cDNA Synthesis Kit (Bio-Rad), including 5 μL 5× iScript Reaction Mix, 1 μL iScript Reverse Transcriptase, and 4 μL nuclease-free water.

### 4.11. Quantitative Real-Time PCR (qRT-PCR)

To determine the quantitative expression of the genes of interest, real-time PCR was used. cDNA was prepared as described above, and primers were obtained from Integrated DNA Technologies, Inc. (IDT, Coralville, IA, USA), and sequences are listed in Table 1. PCR reactions were completed in triplicate using 1 μL of cDNA for each sample. Both forward and reverse primers (1 μL each) for the gene of interest were used in conjunction with 5 μL of iTaq SYBR green (Bio-Rad) and 2 μL of nuclease-free water for a total volume of 10 μL. Once samples were prepared they were used in the CFX Connect ^TM^ Real-Time PCR Detection System (Bio-Rad). Thermal cycling was initiated at 94 °C for 4 min followed by 35 cycles of PCR (94 °C, 15 s; 60 °C, 30 s; 72 °C, 30 s). GAPDH was used as an endogenous control, and vehicle control was used as a calibrator. The relative changes of gene expression were calculated using the following formula: Fold change in gene expression, 2^−ΔΔCt^ = 2 − [ΔCt (treated samples) – ΔCt (untreated control samples)], where ΔCt = Ct (genes of interest) – Ct (GAPDH) and Ct represents threshold cycle number.

### 4.12. miRNA Real-Time PCR

To determine the quantitative expression of miR-221/222, specialized real-time PCR was used. cDNA was prepared using the TaqMan® Advanced miRNA Assay kit (Applied Biosystems, Foster City, CA, USA) and TaqMan® Small RNA Assays (Applied Biosystems). All reactions were performed in triplicate and FAM Supermix (Applied Biosystems) was used in the CFX Connect™ Real-Time PCR Detection System (Bio-Rad). Thermal cycling was initiated at 95 °C for 20 s followed by 40 cycles of PCR (95 °C, 3 s; 60 °C, 30 s). RNU6B was used as an endogenous control [32]. The relative changes of gene expression were calculated using the following formula: Fold change in gene expression in the control, 2^(ΔCt(Untreated samples))^ = X; the experimental samples, 2^(ΔCt(Treated samples))^ = Y; the overall fold change = Y/X, where ΔCt = Ct (gene of interest) – Ct (RNU6B) and Ct represents threshold cycle number. The RNU6B Assay ID is 001093 (Applied Biosystems). miR-221/222 Assay IDs are available in Table 1.

### 4.13. Western Blotting

Western blot of protein extracts is used to determine gene expression at the protein level. For Western blot analysis, protein extracts were prepared as described above (Section 4.9), according to the manufacturer’s protocols. Protein concentration was determined with the Bradford method of protein quantification using the Bio-Rad Protein Assay (Bio-Rad). The same amount (50 μg) of whole cell protein extract was loaded onto a 4–15% Tris-HCL gel (Life Technologies, Carlsbad, CA, USA) and separated by electrophoresis at 200 V until the dye front reaches the bottom of the gel. Separated protein was then transferred to a nitrocellulose membrane using the Trans-Blot Turbo Transfer System (Bio-Rad). Membranes were blocked in 0.5% dry milk in 1× Tris Buffered Saline solution with 0.5% Tween (TBST) using the SNAP i.d. ^®^Protein Detection System (EMD Millipore, Burlington, MA, USA). Primary antibody incubations occurred for 20 min before four 10 mL washes with TBST, and then probing occurred with a secondary antibody for 20 min. Four more washes of 10 mL TBST occurred before incubation in 1.5 mL Clarity^TM^ ECL Western Blotting Substrate (Bio-Rad) was added. The blot was incubated in the dark for 5 min before visualization with the ChemiDoc^TM^ XRS+ System (Bio-Rad) with Image Lab^TM^ software. Antibodies were obtained from Santa Cruz Biotechnology (Dallas, TX, USA) and Cell Signaling Technology.

### 4.14. ChIP qRT-PCR

Chromatin immunoprecipitation (ChIP) was used to determine the quantity of protein localization to a portion of the genome. For ChIP qRT-PCR, cells were fixed, collected, and lysed according to the manufacturer’s protocols (Abcam; Cambridge, MA, USA). Lysed cells were sonicated using a Bioruptor^®^ (Diagenode) to achieve the desired DNA fragment length and validated on a 1.5% agarose gel. Immunoprecipation crosslinked antibodies to regions of the chromatin, while purification isolated only these regions of the DNA according to the manufacturer’s protocols (Abcam). ChIP qRT-PCR primer sequences can be found in Table 2.

### 4.15. Wound Healing Assay

Cells (5 × 10^5^) were seeded on a 6-well plate and given 24 h to adhere. The cells were treated with the indicated compounds at 24 h. At 48 h, a 1 mL pipette tip was used to scratch the monolayer of cells in the center of the well. Another scratch was created perpendicular to the initial scratch. This secondary scratch was used as the point of reference. After scratching, the cells were washed with 1× PBS, and an inverted microscope (Zeiss) was used to take pictures of the cells. Fresh media was added to each well, and the cells were allowed to grow for 8 h in the incubator. At 8 h, another picture was taken using the inverted microscope, and the area migrated was determined using the ImageJ software Wound Healing Tool, where the larger area indicated less migration.

### 4.16. Immunofluorescence

Cells (5 × 10^4^) were seeded on an 8-chambered slide (Millipore) and given 24 h to adhere. The cells were treated with the indicated compounds for three days. The cells were then fixed in 4% paraformaldehyde in 1× PBS and subsequently blocked with 2% FBS, 2% glycine, and 0.5% TWEEN for 30 min. Anti-E-cadherin rabbit monoclonal antibody (Cell Signaling Technology) was applied overnight at a 1:100 dilution with 1.5% goat serum and washed 3 times with PBST. Alexa Fluor® 488 secondary antibody (Abcam) was applied for 30 min at a 1:1000 dilution and washed 3 times with PBST. ProLong™ Gold Antifade Mountant with DAPI (Thermo Fisher Scientific, Grand Island, NY, USA) was added to the chambers before placing the coverslip. Mounting media was allowed to dry overnight before imaging with an EVOS microscope (Thermo Fisher Scientific).

### 4.17. Cell Cycle Analysis

Cells were washed with 1× PBS, fixed in 66% ethanol, and pelleted at 500× *g* for 5 min. The cells were resuspended in 1× propidium iodide + RNase Staining Solution according to the protocol supplied by the Propidium Iodide Flow Cytometry Kit (Abcam). Propidium iodide fluorescence intensity was collected using the UAB Flow Cytometry Core after incubating in the dark at 37 °C for 30 min.

### 4.18. Statistical Analysis

The statistical significance of differences between the values of treated samples and the controls was determined by Students *T*-test using Microsoft Excel. In each case, *p* < 0.05, *p* < 0.01, and *p* < 0.001 were considered statistically significant.

## 5. Conclusions

In summary, SAHA and EGCG are two compounds, synthetic and natural, respectively, that have been shown to be effective in inhibiting cancer cell growth. Despite TNBCs lacking ERα, PR, and HER2, the cell lines used in this study all exhibited different effects of treatment with SAHA and EGCG. Prior studies have not combined SAHA with a dietary phytochemical to explore growth potentiation as explored in the current study. We report decreases in DNMT activity, down-regulation of miR-221/222, and changes in histone acetylation, suggesting epigenetic mechanisms behind the decrease in cellular viability. This down-regulation resulted in an increase in protein levels of p27, ERα, and PTEN. We also report a decrease in cellular migration, an increase in *E-cadherin*, and a decrease in *N-cadherin* with the combination of SAHA and EGCG. We believe that further studies may have translational significance in reducing breast cancer growth and invasion through combining a few cups of green tea each day with traditional chemotherapeutics.

## Figures and Tables

**Table 1:** qRT-PCR primer sequences.

Forward Primer Sequences	Reverse Primer Sequences
GAPDH Sense: 5′-ACCACAGTCCATGCCATCAC-3′	GAPDH Anti-Sense: 5′-TGCACCCTGTTGCTGTA-3′
miR-221 Sense: Assay ID: 478778_mir	miR-221 Anti-Sense: Assay ID: 477981_mir
miR-222 Sense: Assay ID: 478779_mir	miR-222 Anti-Sense: Assay ID: 477982_mir
p27 Sense: 5′-GTTAACCCGGGACTTGGA-3′	p27 Anti-Sense: 5′-CACCTCTTGCCACTCGTA-3′
ERα Sense: 5′-GAACCGTCCGCAGCTCAAGATC-3′	ERα Anti-Sense: 5′-GTCTGACCGTAGACCTGCGCGTTG-3′
PTEN Sense: 5′-CAAGATGATGTTTGAAACTATTCCAATG-3′	PTEN Anti-Sense: 5′-CCTTTAGCTGGCAGACCACAA-3′
E-cadherin Sense: 5′-CGGAGAAGAGGACCAGGACT-3′	E-cadherin Anti-Sense: 5′-GGTCAGTATCAGCCGCTTTC-3′
N-cadherin Sense: 5′-ACAGTGGCCACCTACAAAGG-3′	N-cadherin Anti-Sense: 5′-CCGAGATGGGGTTGATAATG-3′

**Table 2:** ChIP qRT-PCR primer sequences.

Forward Primer Sequences	Reverse Primer Sequences
p27 Sense: 5′-CTGAGCGAACCATTGCCCA-3′	p27 Anti-Sense: 5′-AACAAACTAGCCAAACGGCC-3′
PTEN Sense [19]: 5′-CAGACTTGACAGGTTTGTTC-3′	PTEN Anti-sense [19]: 5′-TCCAGTCACTACCCCTGAGC-3′

## Supplementary Materials

The following are available online at http://www.mdpi.com/2072-6694/11/1/23/s1, Figure S1: SAHA and EGCG significantly reduce cellular viability in three triple-negative and one ER-positive breast cancer cell lines. All cell lines were treated for 72 h with SAHA and EGCG, alone or in combination. There were no significant changes in cellular viability in the MCF10A non-cancerous control cells with low single doses of SAHA or EGCG (A). ER-positive MCF-7 cells (B) and all three triple-negative cell lines (C–E) experienced a reduced viability with the combination of SAHA and EGCG. We chose to proceed with 3 μM SAHA and 5 μM EGCG. Error bars represent standard error of the mean (SEM); * *p* < 0.05, ** *p* < 0.01, *** *p* < 0.001, Figure S2: SAHA and EGCG increase G1/S phase in three of four breast cancer cell lines. (A) MCF-7 cells were treated with SAHA and/or EGCG for 72 h prior to FACS analysis. Cell cycle analysis was performed using propidium iodide in the MCF-7 cell line. (B–D) The same was done in the MDA-MB-157, MDA-MB-231, and the HCC1806 TNBC cell lines. All experiments were repeated three times. Error bars represent standard error of the mean (SEM); * *p* < 0.05, ** *p* < 0.01, *** *p* < 0.001.
