# Supplementary material for: Effects of SAHA and EGCG on Growth Potentiation of Triple-Negative Breast Cancer Cells
Source: Cancers (Basel). 2018 Dec 27;11(1):23. doi: 10.3390/cancers11010023 (PMC6356328; doi:10.3390/cancers11010023)
Supplement: Supplementary file 1 [file cancers-11-00023-s001.pdf]

Supplementary materials: Effects of SAHA and EGCG on Growth Potentiation of Triple-Negative Breast Cancer Cells

Kayla A. Lewis, Harrison R. Jordan and Trygve O. Tollefsbol

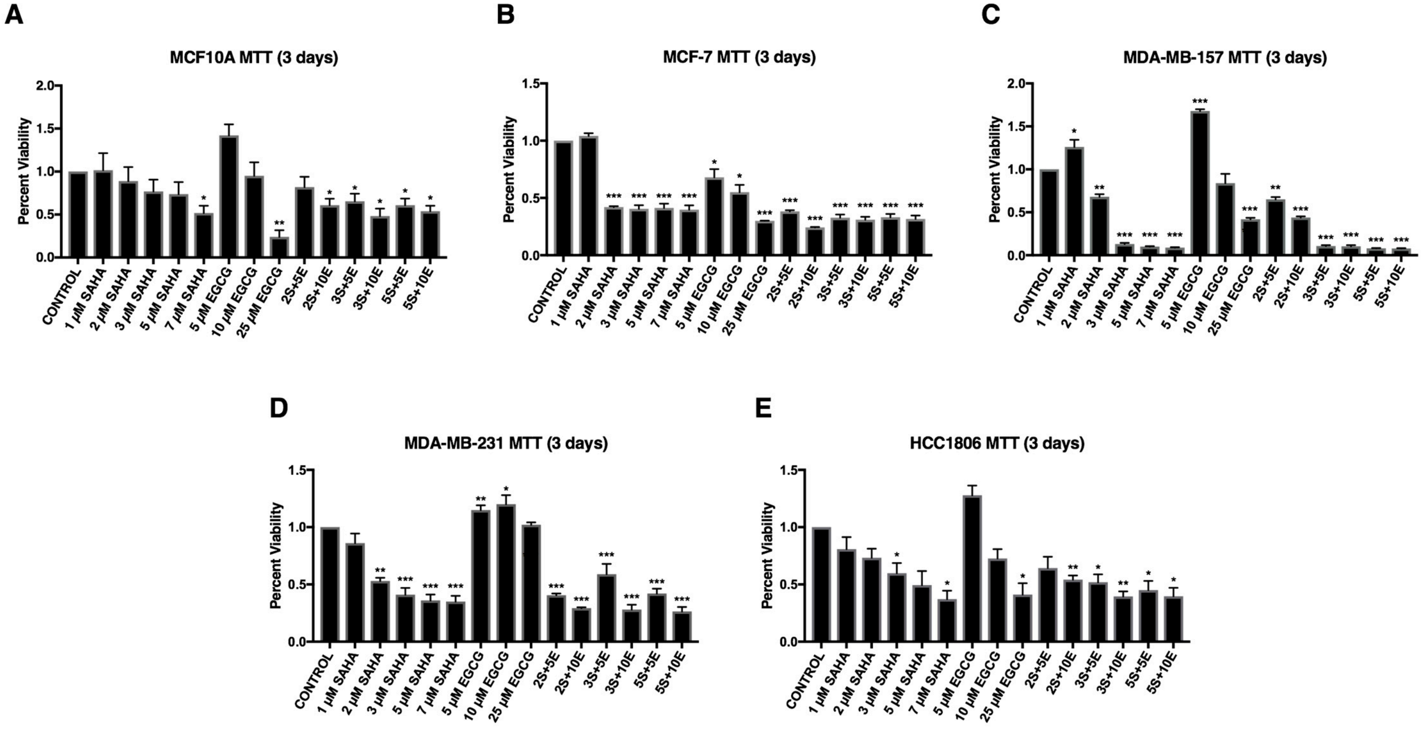

Figure S1. SAHA and EGCG significantly reduce cellular viability in three triple-negative and one ER-positive breast cancer cell lines. All cell lines were treated for 72 h with SAHA and EGCG, alone or in combination. There were no significant changes in cellular viability in the MCF10A non-cancerous control cells with low single doses of SAHA or EGCG (**A**). ER-positive MCF-7 cells (**B**) and all three triple-negative cell lines (**C–E**) experienced a reduced viability with the combination of SAHA and EGCG. We chose to proceed with 3 μM SAHA and 5 μM EGCG. Error bars represent standard error of the mean (SEM); * $p < 0.05$, ** $p < 0.01$, *** $p < 0.001$.

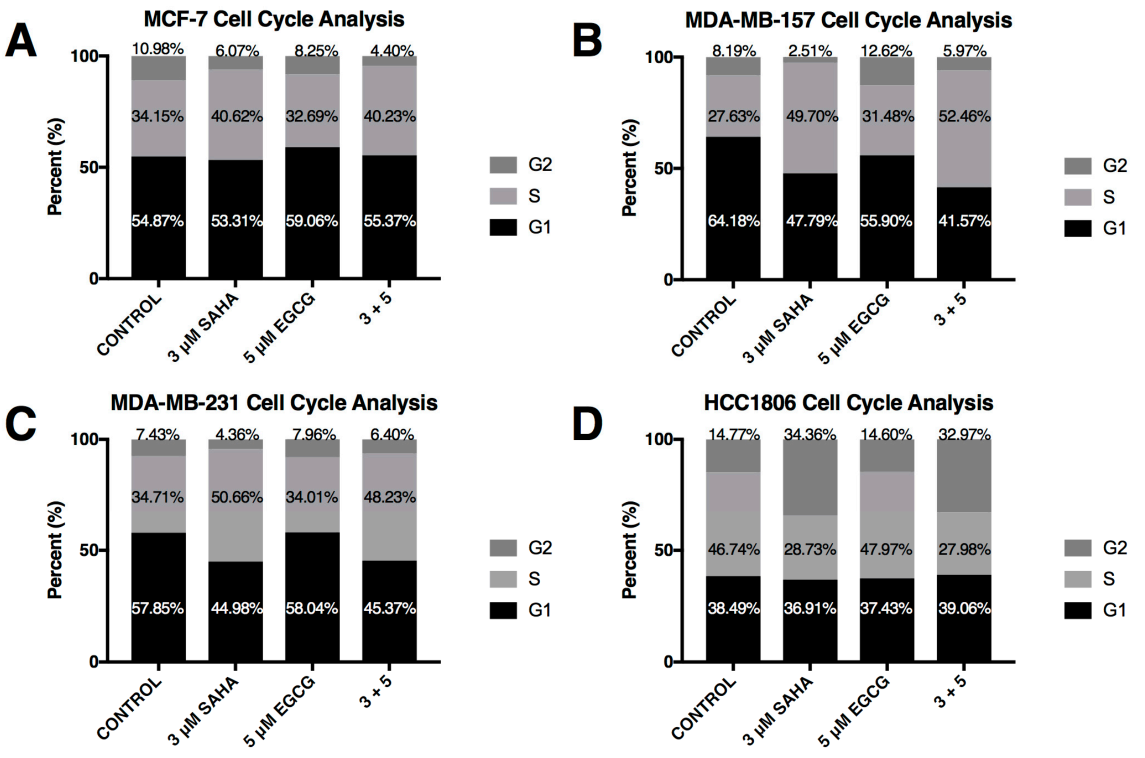

Figure S2. SAHA and EGCG increase G1/S phase in three of four breast cancer cell lines. (**A**) MCF-7 cells were treated with SAHA and/or EGCG for 72 h prior to FACS analysis. Cell cycle analysis was performed using propidium iodide in the MCF-7 cell line. (**B**–**D**) The same was done in the MDA-MB-157, MDA-MB-231, and the HCC1806 TNBC cell lines. All experiments were repeated three times. Error bars represent standard error of the mean (SEM); * $p < 0.05$, ** $p < 0.01$, *** $p < 0.001$.

© 2018 by the authors. Licensee MDPI, Basel, Switzerland. This article is an open access article distributed under the terms and conditions of the Creative Commons Attribution (CC BY) license (http://creativecommons.org/licenses/by/4.0/).
